# Basement Membrane-Associated lncRNA Risk Model Predicts Prognosis and Guides Clinical Treatment in Clear Cell Renal Cell Carcinoma

**DOI:** 10.3390/biomedicines11102635

**Published:** 2023-09-26

**Authors:** Xinxin Li, Qihui Kuang, Min Peng, Kang Yang, Pengcheng Luo

**Affiliations:** 1 Department of Urology, Wuhan Third Hospital (Tongren Hospital of Wuhan University), Wuhan 430060, China; xinxinli0408@whu.edu.cn (X.L.); 2021283030166@whu.edu.cn (Q.K.); 2 Department of Oncology, Renmin Hospital of Wuhan University, Wuhan 430060, China; 3 Department of Urology, Renmin Hospital of Wuhan University, Wuhan 430060, China; kangyang@whu.edu.cn

**Keywords:** basement membrane, diagnostic, renal cancer, lncRNA, prognostic model, biomarker, immunotherapy

## Abstract

The basement membrane (BM) affects the invasion and growth of malignant tumors. The role and mechanism of BM-associated lncRNAs in clear cell renal cell carcinoma (ccRCC) are unknown. In this study, we identified biomarkers of ccRCC and developed a risk model to assess patient prognosis. We downloaded transcripts and clinical data from the Cancer Genome Atlas (TCGA). Differential analysis, co-expression analysis, Cox regression analysis, and lasso regression were used to identify BM-associated prognostic lncRNAs and create a risk prediction model. We evaluated and validated the accuracy of the model using multiple methods and constructed a nomogram to predict the prognosis of ccRCC. GO, KEGG, and immunity analyses were used to explore differences in biological function. We constructed a risk model containing six BM-associated lncRNAs (LINC02154, IGFL2-AS1, NFE4, AC112715.1, AC092535.5, and AC105105.3). The risk model has higher diagnostic efficiency compared to clinical characteristics and can be used to forecast patient prognoses. We used renal cancer cells and tissue microarrays to verify the expression of lncRNAs in the risk model. We found that knocking down LINC02154 and AC112715.1 could inhibit the invasion ability of renal cancer cells. The risk model based on BM-associated lncRNAs can well predict ccRCC and guide clinical treatment.

## 1. Introduction

As the most prevalent form of kidney cancer, ccRCC is highly aggressive and metastatic [1]. Approximately 80% of all renal cell carcinomas are clear cell renal cell carcinomas (ccRCCs) [2], which are the second most frequent kind of urinary system cancer, behind bladder cancer. Approximately one third of ccRCC patients may have metastases at the time of their first diagnosis, and one fourth of individuals with localized cancer may experience disease recurrence after a complete surgical excision [3]. A significant death rate is often linked with metastatic ccRCC [4,5]. Even with surgery, chemotherapy, radiation therapy, targeted therapy, and the newly suggested immunotherapy, ccRCC remains one of urology’s greatest clinical difficulties [6]. A late diagnosis and a high risk of metastases are the primary causes [7]. Given the significant mortality and morbidity associated with ccRCC, it is essential to identify effective therapeutic targets, develop more accurate prognostic models, and identify relevant biomarkers for ccRCC patients.

The basement membrane (BM) is a thin, dense extracellular matrix (ECM) layer that is essential for the formation and function of normal tissues [8]. The BM contains an abundance of biochemical and mechanical signals and is necessary for cell signaling, structural integrity, and barrier protection against cells and macromolecules. Cancer is linked to changes in the mechanical and chemical properties of the BM [9]. As a protective structural barrier that impedes the invasion, migration, and extravasation of cancer cells, the BM plays an essential function in epithelial carcinomas and carcinomas. To metastasize, cells must enter through the basement membrane, which is a physical barrier preventing cancer cells from invading the surrounding stromal tissue. The endothelial BM impedes the invasion (intraluminal) and outflow (extravasation) of blood and lymphatic vessels by cancer cells during metastasis, which is associated with 90% of cancer deaths [10]. The growth of metastases or malignancies is a significant obstacle for cancer therapy and is a key factor contributing to higher mortality. The 5-year survival rate reduces considerably after cells enter the surrounding region via the BMS [11]; BM integrity is a crucial prognostic indication for patients. A thorough understanding of BM structure and processes, as well as cancer cell invasion of the BM, may lead to the development of innovative techniques for inhibiting cancer growth and metastasis.

RNAs longer than 200 base pairs (bps) are called long non-coding RNA (lncRNA), which do not have protein-coding activity and have an essential role in the regulation of the immune response. It is associated with immune cell infiltration, tumor elimination, antigen recognition, and exposure [12]. According to recent research, lncRNAs are involved in various tumor development pathways, encompassing carcinogenesis, proliferation, migration, invasion, and metastasis, as well as angiogenesis [13,14]. Liu demonstrated that lncRNAs participate in tumor autophagy [15]. Numerous studies have revealed that lncRNAs may influence target gene expression by competing with target genes [12,16,17]. LncRNAs are also associated with tumor therapy resistance [18]. However, the functions of basement membrane-associated lncRNAs in the prognosis of ccRCC and tumor immunotherapy remain unknown. This work aimed to build a prognostic risk model of basement membrane-associated lncRNAs to assess prognosis and guide clinical treatment in clear cell renal cell carcinoma.

## 2. Materials and Methods

### 2.1. Collect and Identify lncRNAs Connected with Basement Membranes

From the TCGA database, we collected clinical and transcriptome information for 541 ccRCC patients (https://portal.gdc.cancer.gov/repository (accessed on 1 December 2022)). Patients with inadequate clinical data were eliminated from the study. By doing a literature search, genes associated with basement membranes were found [19]. We screened for basement membrane genes and lncRNAs with differential expression between normal and renal clear cell carcinoma tissues using a *p*-value of 0.05 and |log2FC| > 1.5 as cutoff values. We then identified lncRNAs linked with differentially expressed basement membrane genes using Pearson correlation analysis (|correlation coefficient| > 0.60, *p* < 0.001).

### 2.2. Construction and Verification of a Basement Membrane-Associated lncRNA Risk Model

The ccRCC patient data were assigned randomly to either the training set or the testing set in a ratio of 1:1. The training set was used to construct a risk model for basement membrane-associated lncRNAs, whereas the testing set and the overall set were utilized to verify the risk model. Basement membrane-associated lncRNAs linked with kidney carcinoma prognosis were identified using univariate Cox regression analysis. A prognostic risk model based on the optimal lncRNA was developed by utilizing the LASSO Cox regression technique and multivariate Cox regression analysis. Using this risk model, a risk score was assigned to every individual. This is how the risk score is computed: risk score = ∑ i = 1nCoef(i) × Expr(i). In the equation, Coef (i) represents the regression coefficient of each lncRNA, and Expr (i) represents the normalized expression level of each lncRNA. Using the median risk score, the training set was divided into low- and high-risk groups. We applied Kaplan–Meier curves to explore if the two risk groups differed in terms of overall survival. We drew receiver operating characteristic (ROC) curves for clinical characteristics and prognostic models, evaluated the area under the curve (AUC), and used the concordance index (C-index) to evaluate the risk model’s accuracy.

### 2.3. Creating and Validating Predicted Nomograms and Evaluating the Relationship between the Prognostic Signatures and Clinicopathological Features

To predict OS in ccRCC patients at 1, 3, and 5 years, we built nomograms using the rms R package (R 4.2.1) based on clinical parameters and risk scores. According to the nomogram scoring technique, each variable is given a score, and the total score for each sample is calculated by summing the scores of every variable. The prediction ability of an existing nomogram model was evaluated using a nomogram calibration plot. The connection between basement membrane-associated lncRNAs and clinicopathological characteristics was studied using logistic regression and heat maps.

### 2.4. PCA, Functional Enrichment, Tumor Immunity, Drug Sensitivity, and Mortality Analysis

Principal component analysis (PCA) was used to investigate the geographical distribution of two risk groups across four expression profiles (total gene expression profile, basement membrane gene expression profile, basement membrane-associated lncRNA expression profile, and six basement membrane-associated lncRNA expression profiles in the risk model). We evaluated enrichment pathways and biological processes for genes that were expressed differently in two risk groups using GO and KEGG. The enrichment of biological processes and pathways was highly significant only when *p* < 0.05 and FDR <0.05.

The dataset of tumor immune cells was obtained using TIMER 2.0 (http://timer.cistrom.org (accessed on 5 December 2022)). We used seven algorithms to simultaneously compare differences in the immune infiltration profile between the two risk groups (TIMER, CIBERSORT, CIBERSORT-ABS, QUANTISEQ, MCPCOUNTER, XCELL, and EPIC). We employed heatmaps to illustrate variations in immune infiltration status under varied algorithmic conditions. Additionally, single-sample GSEA (ssGSEA) was performed to evaluate immune-related functions of ccRCC, and a heat map was shown. We collated previous studies to identify potential immune checkpoints and assess expression differences between the two risk groups [20]. These stages used the R packages limma, pHeatmap, ggpubr, GSEABase, reshape2, and ggplot2. We obtained the tumor immune dysfunction and exclusion (TIDE) scoring result of every ccRCC patient from the TIDE database (http://tide.dfci.harvard.edu (accessed on 10 December 2022)).

We used the “ggpubr” tool to compare the immune checkpoint blockade (ICB) responses of the two risk populations. Subsequently, we used the pRRophetic program to predict the medicines that may be used to treat ccRCC and to estimate the IC50 values of the pharmaceuticals in the two patient groups. We assessed the relationship between patient mortality and risk score by calculating the proportion of patients who died in the two risk groups and the risk score for dead and surviving patients. In addition, we also compared whether there was a significant difference in progression-free survival (PFS) between the two risk groups of patients.

### 2.5. Validation of Basement Membrane-Associated lncRNA Expression in Renal Cancer Cells

Bena Culture Collection (BNCC, Beijing, China) provided the normal kidney cell line (HK-2, RRID: CVCL_0302) and the renal cancer cell lines (ACHN, RRID: CVCL_1067; 769-P, RRID: CVCL_1050; and CAKI-1, RRID: CVCL_0234). All experimental cells were free of mycoplasma contamination via the assay (Service-bio, #G1900-50T, Wuhan, China). The cells were grown in a 10% fetal bovine serum-containing DMEM/F-12 mixture. All cells were cultivated at 37 °C and 5% CO_2_ in the incubator. We used the TRIzol reagent to extract total RNA from cells and tissues (Servicebio, #G3013, Wuhan, China). Finally, cDNA was treated to qRT-PCR using SYBR Green qPCR Master Mix (Servicebio, #G3320-01, Wuhan, China). GAPDH served as the internal benchmark. We used GraphPad Prism 8 (San Diego, CA, USA) for statistical analysis, and various group means were compared using one-way ANOVA. Below are the primer sequences we used: LINC02154-F: ACCAATGAGACAATGCCACTGAACC; LINC02154-R: TGACCC CTGATTGTGCCTGAAAG; IGFL2-AS1-F: TGGTCTAGCGGTAGCGTCAGTG, IGFL2-AS1-R: ACAAGAGGTGGTGGAGC AGAGTC; NFE4-F: GGGGTGGGCATGTGTTGACTT, NFE4-R: GCTGGAGTGTGG ATGGTG GAAAC; AC112715.1-F: GCTGCTGTGCTGACCAGTCTG, AC112715.1-R: CTTGGTGGAATG GCAGGAAGAGC; AC092535.5-F: GCCACACTTGCCTTCCTGCTG, AC092535.5-R: CCTG TCC ACTTGCCTGTTGCC; and AC105105.3-F: TGGCACTCCTGGAGCACTCTG, AC105105.3-R: TGTA GGACGCTATGGCTGGGAAG.

### 2.6. RNA Fish

Clear cell renal cell carcinoma tissue chips were purchased from Shanghai Outdo Biotech Co., Ltd., Shanghai, China, (#HKid-CRCC060PG-01). The tissue chips were deparaffinized, fixed with 4% paraformaldehyde, and permeabilized. Design RNA probes are complementary to the target RNA sequence (lncRNA LINC02154: TTAGTGGCTTCTCCCCACAGTGAAC, AAAGCCACGACACAATCAAAACCTC, TTGACCCACTGATTGTGCCTGAAAG; lncRNA AC112715.1: ACTGAAACTTCCGTGGTAGGTGGCT, CAAAACGGGACTC CACCTTGACATC, AAGAACCAGGCAATCCTTTGTCTCC, TTAGCAATGAA GTC TCGGATGGCAT; lncRNA AC092535.5: AACCACCACCTCATCAACGACTTCA, TGTCT GCCT GTGTTCTCTTCCTCCC, GACCAGTCCGTTTGACACTGAGTGG, TGTGGAAGAGAATGGCAGAGACAGA; and lncRNA AC105105.3: AGGAAACTCTGTAGCCACGAAGGTG, GCAAACGATGCCAAGACATTTATCG, GCTGGGAAGAAA CAGTGAGAGGTGA, GAGGGAAGGATTGCCTAGCAGTAGC). The probe was labeled with Cy3 fluorescent dye. Labeled RNA probes were prepared in formamide hybridization buffer to maintain probe stability and facilitate hybridization. Fixed and permeabilized tissue was also incubated in hybridization buffer. The sample was washed multiple times with wash buffer to remove the unbound probe and reduce the background signal. Samples were coated with appropriate mounting medium and coverslips, and images of labeled RNA molecules were captured using a fluorescence microscope (BX41; Olympus, Tokyo, Japan).

### 2.7. Invasion Assay

We transferred small interfering RNA and negative control RNA of two risk model lncRNAs (AC112715.1 and LINC02154) into ACHN and 769P cell lines. After twenty-four hours in serum-free medium, 1 × 105 cells were grown in a 24-well transwell plate with Matrigel for the invasion assay. The transwell plate was placed in a 37 °C, 5% CO_2_ cell culture incubator for 24 h. All cells were treated with 4% paraformaldehyde and crystal violet after twenty-four hours incubation. A random selection of five fields from each slide was randomly utilized for statistical analysis.

## 3. Results

### 3.1. Construction of a Basement Membrane-Associated lncRNA Signature

**Figure 1:**
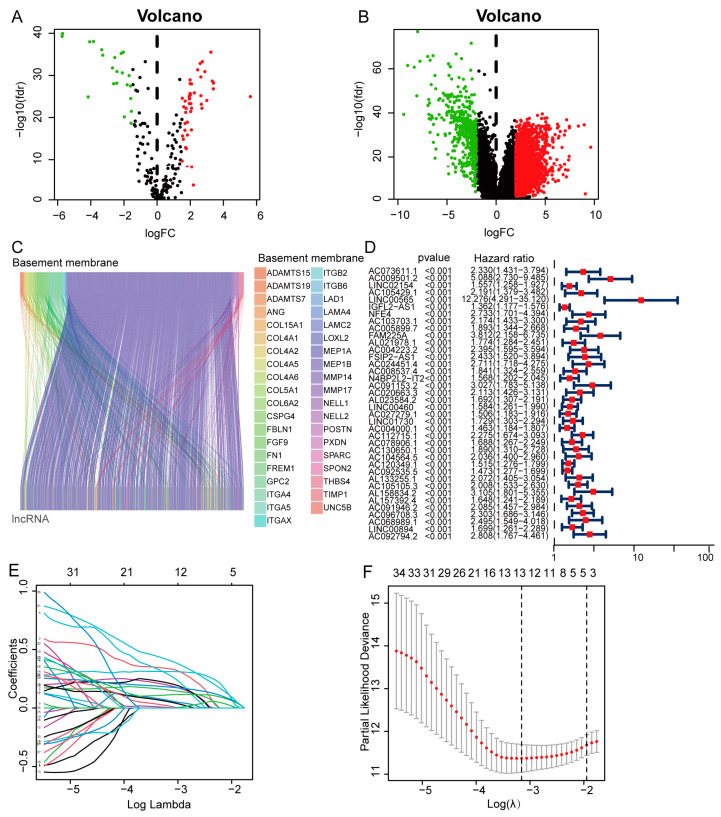
Identification of basement membrane-associated ccRCC prognostic lncRNAs. (**A**) Differential expression of basement membrane genes in ccRCC. (**B**) Differential expression of lncRNAs in ccRCC. (**C**) Sankey relationship diagram of basement membrane genes and basement membrane-associated lncRNAs. (**D**) Basement membrane-associated ccRCC prognostic lncRNAs. (**E**) The least absolute shrinkage and selection operator (LASSO) algorithm’s 10-fold cross-validation of variable selection. (**F**) Distribution of LASSO coefficients of basement membrane-associated lncRNAs.

We analyzed normal and cancer tissues for 68 basement membrane-related genes with differential expression (Figure 1A) and 2573 lncRNAs with differential expression (Figure 1B). Correlation analysis revealed 784 differential (Pearson R > 0.60, *p* < 0.001) basement membrane-associated lncRNAs (Figure 1C). We discovered 38 basement membrane-related lncRNAs linked with patient prognosis using univariate Cox regression (Figure 1D). Next, we performed LASSO-Cox regression on the training set to reduce multicollinearity and found 13 lncRNAs (Figure 1E,F). Subsequent multivariate Cox analysis revealed a risk model comprising six basement membrane-associated lncRNAs (LINC02154, IGFL2-AS1, NFE4, AC112715.1, AC092535.5, and AC105105.3). Next, we explain how the expression levels of basement membrane-associated lncRNAs may be utilized to generate a risk score. The risk score for each patient is as follows: risk score = (LINC02154*0.2227 + IGFL2-AS1*0.3000 + NFE4*0.4607 + AC112715.1*0.5587 + AC092535.5*0.2509 + AC105105.3*0.7305).

### 3.2. Expression and Survival Analysis of Prognostic Model lncRNAs

**Figure 2:**
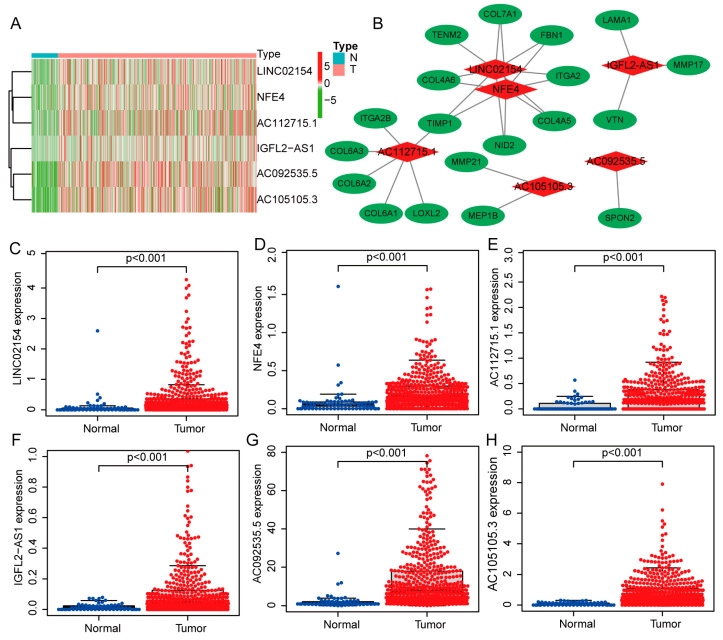
Expression levels and lncRNA-mRNA network of six basement membrane-associated lncRNAs. (**A**) A heat map of the lncRNA expression levels in the risk model. N, normal; T, tumor. (**B**) The co-expression network of prognostic basement membrane-associated lncRNAs. (**C**–**H**) The expression levels of six basement membrane-associated lncRNAs in tumor and normal tissues.

**Figure 3:**
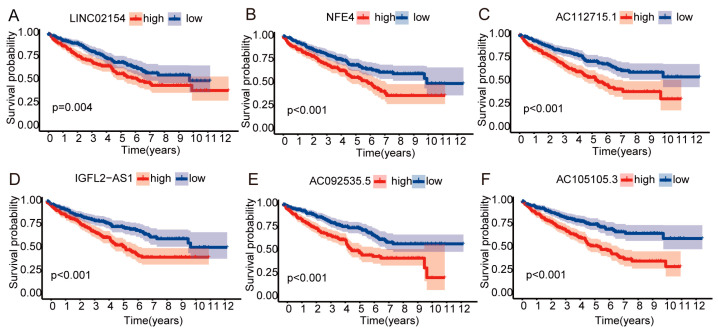
Overall survival of six basement membrane-associated lncRNAs. (**A**) LINC02154, (**B**) NFE4, (**C**) AC112715.1, (**D**) IGFL2-AS1, (**E**) AC092535.5, and (**F**) AC105105.3.

A heat map shows how much six lncRNAs linked to the basement membrane are expressed in patients with ccRCC. All six of these lncRNAs are highly expressed in renal cancer tissues (Figure 2A). We further visualized lncRNAs using the ggalluvial R package (R 4.2.1) and Cytoscape software (3.9.1). The co-expression network included 27 lncRNA-mRNA pairings (Figure 2B, |correlation coefficient| > 0.6 and *p* < 0.001). LINC02154 was co-expressed with eight basement membrane-associated genes (COL4A5, COL4A6, COL7A1, FBN1, NID2, TIMP1, ITGA2, and TENM2), IGFL2-AS1 was co-expressed with three basement membrane-associated genes (LAMA1, MMP17, and VTN), NFE4 was co-expressed with seven basement membrane-associated genes (COL4A5, COL4A6, COL7A1, FBN1, NID2, TIMP1, and ITGA2), AC112715.1 was co-expressed with six basement membrane-associated genes (COL6A1, COL6A2, COL6A3, LOXL2, TIMP1, and ITGA2B), AC092535.5 was co-expressed with SPON2, and AC105105.3 was co-expressed with MEP1 and BMMP21. Figure 2C–H display the expression levels of six lncRNAs associated with the basement membrane in ccRCC patients. They are all highly expressed in cancer tissues, and individuals with high expression of these lncRNAs had shorter overall survival (OS) (Figure 3A–F).

### 3.3. Survival Results and Multivariate Analysis

**Figure 4:**
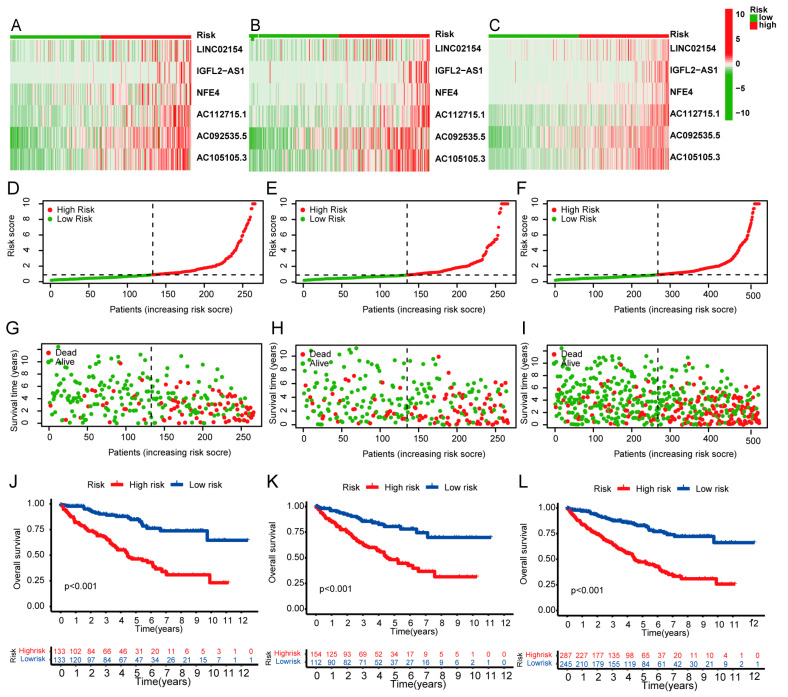
Prognostic analysis of the risk model in training, testing, and overall groups. (**A**–**C**) The clustering analysis heatmap depicts the six lncRNA expression levels for each patient in training, testing, and overall sets. (**D**–**F**) Distribution of basement membrane-associated lncRNA model-based risk score in training, testing, and overall sets. (**G**–**I**) Patterns of survival time and survival status were ranked by risk score in training, testing, and entire sets. (**J**–**L**) Kaplan–Meier survival curves of overall survival of patients in training, testing, and overall sets.

To examine the validity and reliability of the risk model, we also used the median risk score to classify patients in the testing and all sets into two risk groups. Figure 4A–C depict the expression patterns of six basement membrane-associated lncRNAs. Each of the six lncRNAs associated with the basement membrane was enriched in the high-risk population; the risk scores are depicted in Figure 4D–F, and the patient’s survival status is depicted in Figure 4G–I. Clearly, the incidence of ccRCC patient fatalities increased as the risk score rose, and the low-risk category had longer survival times (Figure 4J,K).

**Figure 5:**
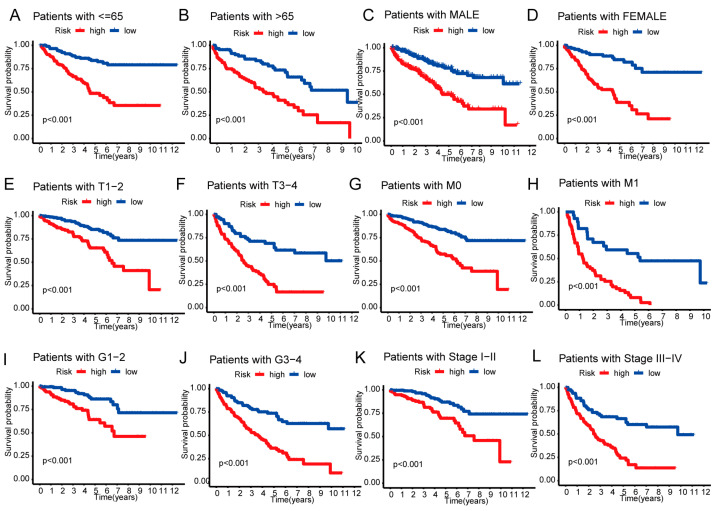
Kaplan–Meier survival curves for low- and high-risk populations according to various clinical factors. (**A**,**B**) age. (**C**,**D**) gender. (**E**,**F**) T stage. (**G**,**H**) M stage. (**I**,**J**) grade. (**K**,**L**) TMN stage.

We analyzed whether there is a difference in survival between the two risk groups at various clinical stages (age, gender, grade TMN stage, T stage, and M stage). Statistically, the low-risk category had a much greater overall survival rate than the high-risk category (Figure 5A–L). The findings imply that the risk model may be applied to evaluate the survival of ccRCC patients with various clinicopathological characteristics.

### 3.4. Independent Prognostic Value of the Risk Score

**Figure 6:**
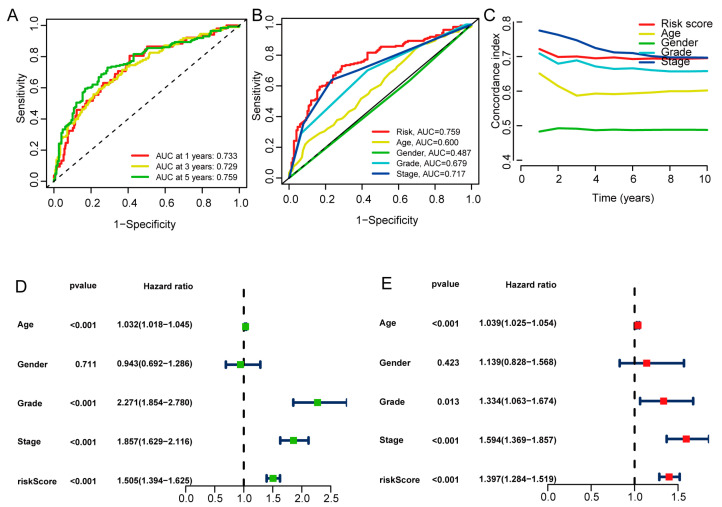
Assessment of the prognostic signature of the basement membrane-associated lncRNAs in the overall set. (**A**) The 1-year, 3-year, and 5-year ROCs of the risk model. (**B**) The 5-year ROC curve of the risk model and clinicopathological variables. (**C**) The C-index curve of the risk model. (**D**) Forest plot for univariate Cox regression analysis. (**E**) Forest plot for multivariate Cox regression analysis.

The 1-, 3-, and 5-year ROCs had corresponding AUC values of 0.733, 0.729, and 0.759 (Figure 6A). In the 5-year ROC curve of the model, the AUC of the risk score was 0.759, displaying more prominent predictive power than other clinicopathological features (Figure 6B). The risk model’s 10-year C-index was similarly high in all aspects (Figure 6C). Using univariate and multivariate Cox regression analyses, age, grade, stage, and the risk model for six basement membrane-associated lncRNAs were identified as independent predictive variables for ccRCC (Figure 6D,E). These results demonstrate the excellent predictive power of the risk model.

### 3.5. Nomogram and Heatmap of Clinical Factors

**Figure 7:**
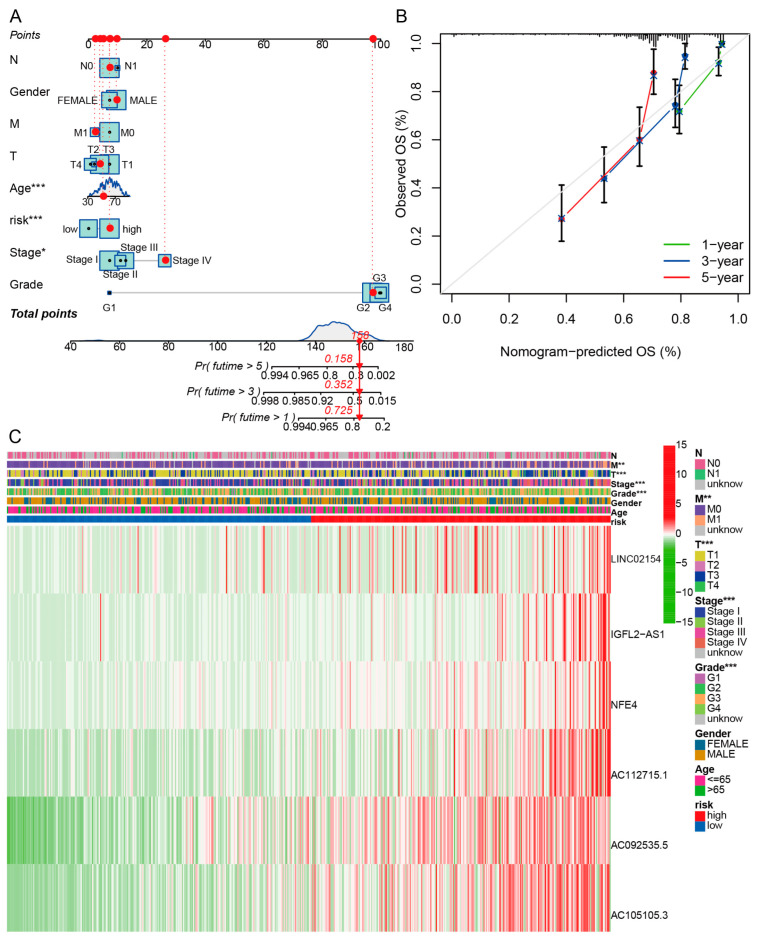
Nomograms and heatmaps of risk scores and clinicopathological features. (**A**) Nomogram of clinicopathological factors and risk scores. (**B**) Calibration curves for detecting nomogram predictions. (**C**) Heatmap for basement membrane-associated lncRNAs prognostic signature and clinicopathological variables. * *p* < 0.05; ** *p* < 0.01; *** *p* < 0.001.

We evaluated the outcomes of patients with ccRCC at 1, 3, and 5 years using a nomogram comprising clinicopathological features and the risk score (Figure 7A). The calibration curve shows that the predicted result of the nomogram and the actual result are very close (Figure 7B). A heatmap depicting the relationship between the prediction signature of basement membrane-associated lncRNAs and clinicopathological characteristics was also drawn (Figure 7C). The risk score was related to T stage, M stage, TMN stage, and grade; patients with higher risk scores tended to have a higher clinical stage.

### 3.6. PCA and Enrichment Analysis

**Figure 8:**
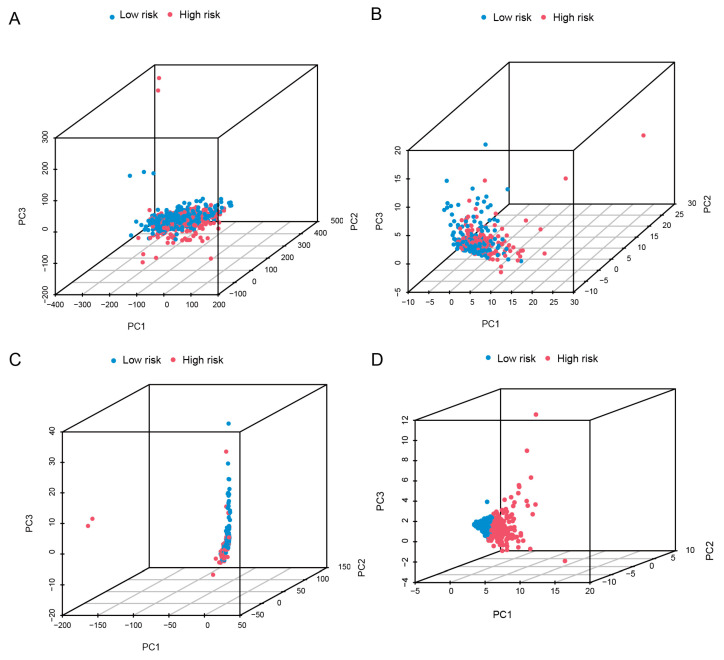
Principal component analysis between the high- and low-risk populations. (**A**) PCA of all genes. (**B**) PCA of basement membrane genes. (**C**) PCA of basement membrane-associated lncRNAs. (**D**) PCA of risk lncRNAs.

We examined the spatial distribution of two risk groups across four expression profiles using PCA (total gene expression profile, basement membrane gene expression profile, basement membrane-associated lncRNA expression profile, and six basement membrane-associated expression profiles in the risk model) (Figure 8A–D). The results suggested that six basement membrane-associated lncRNAs had the greatest potential to differentiate between populations at low and high risk.

**Figure 9:**
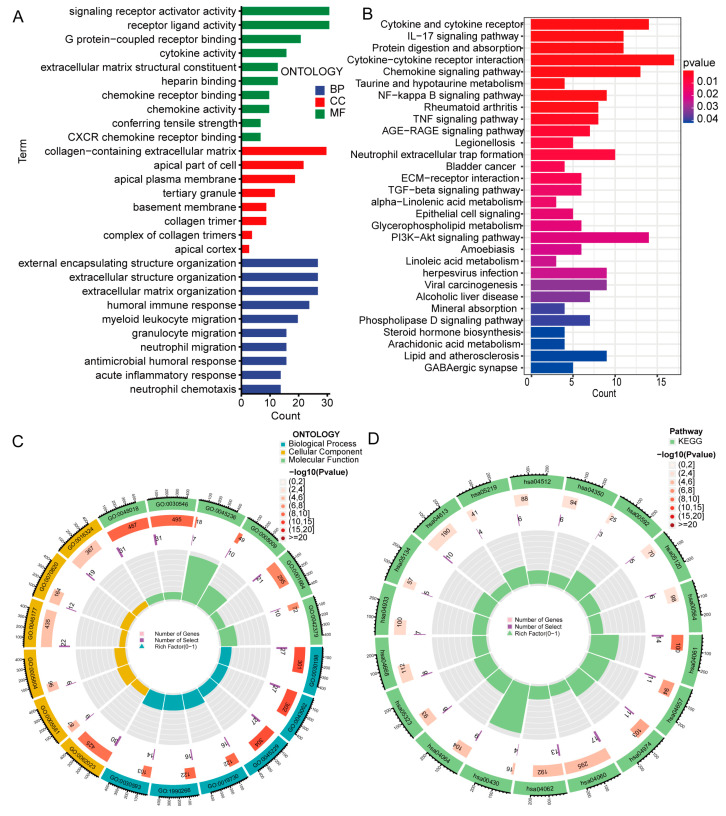
GO and KEGG analysis of DEGs in high- and low-risk groups. (**A**,**C**) GO analysis of DEGs. (**B**,**D**) KEGG analysis of DEGs. GO, gene ontology; KEGG, Kyoto Encyclopedia of Genes and Genomes; DEGs, differentially expressed genes; BP, biological process; CC, cellular component; MF, molecular function.

The basement membrane-associated lncRNAs were substantially connected with extracellular matrix structure and inflammatory cell motility, according to GO analysis (Figure 9A,C). The KEGG study resulted mainly in cytokine receptor and IL-17 signaling route; cancer-related signaling pathways including NF-kappa B, TGF-β, TNF, PI3K-Akt; and the viral carcinogenesis signaling pathway (Figure 9B,D). These results suggest that basement membrane-associated lncRNA plays an important role in the development of clear cell renal cell carcinoma.

### 3.7. Examination of Immune Characteristics Using the Basement Membrane-Related lncRNA Signature

**Figure 10:**
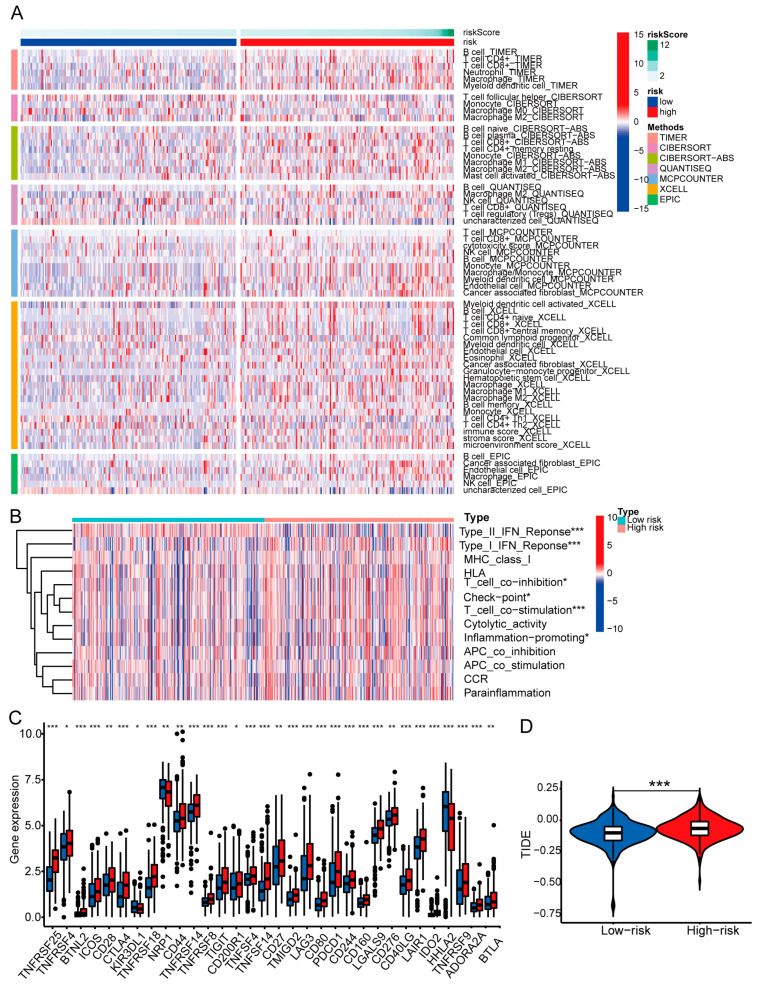
Differences in the tumor immune microenvironment between the low- and high-risk groups. (**A**) Heatmap for immune cell infiltration landscape between the two risk groups. (**B**) ssGSEA scores of immune cells and immune function in the two risk groups. (**C**) Expression of immune checkpoints between high- and low-risk groups. (**D**) TIDE scores between the two groups. * *p* < 0.05; ** *p* < 0.01; *** *p* < 0.001.

The heatmap in Figure 10A displays immune cell infiltration based on seven algorithms. We examined the association between risk score and immune-related response. The data show that type II IFN response, type I IFN response, T cell co-inhibition, checkpoints, T cell co-stimulation, and inflammatory promotion are differently active across two risk groups (Figure 10B). Moreover, we compared immune checkpoint alterations between the two risk groups (Figure 10C). The majority of immunological checkpoints were expressed at higher levels in the high-risk group, which may explain patients’ shorter survival time. The high-risk population had higher TIDE scores, suggesting reduced responses and shorter survival in ICI-treated patients, which may explain their poor prognoses (Figure 10D).

### 3.8. Therapeutic Drug Sensitivity and Mortality Rate

**Figure 11:**
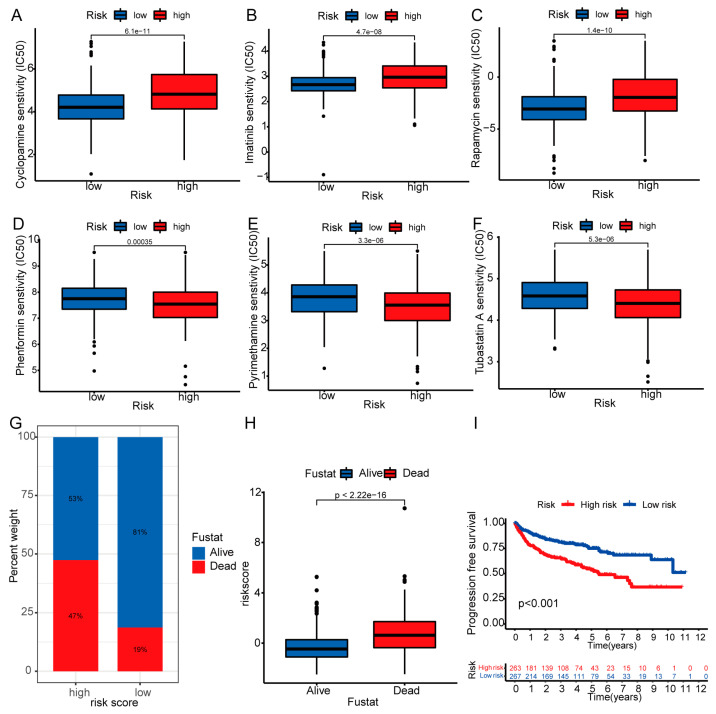
Differences in drug sensitivity and prognoses between high- and low-risk groups. Sensitive drugs in low-risk groups: (**A**) cyclopamine, (**B**) imatinib, and (**C**) rapamycin; Sensitive drugs in high-risk groups: (**D**) phenformin, (**E**) pyrimethamine and (**F**) tubastatin. (**G**) The difference in the proportion of dead patients between the two risk groups. (**H**) The differences in risk scores between dead and alive patients. (**I**) The difference in PFS of patients in the two risk groups.

We discovered considerable disparities in IC50 values for various medicines between the two patient groups by evaluating their drug susceptibilities. Cyclopamine, imatinib, and rapamycin were more efficient in low-risk populations (Figure 11A–C), whereas phenformin, pyrimethamine, and tubastatin A were more efficient in high-risk populations (Figure 11D–F). These results suggest that risk scores may be useful in guiding clinical treatment. By comparing the percentage of patients who died in the two risk categories, we determined that the high-risk group had a larger percentage of deceased patients (Figure 11G). Patients who died had a much higher risk score than survivors (Figure 11H). Moreover, we analyzed the progression-free survival (PFS) of patients in the two risk categories and found that PFS durations were shorter for the high-risk categories (Figure 11I). These results suggest that risk models can predict patient mortality.

### 3.9. Expression of Risk Model lncRNAs

**Figure 12:**
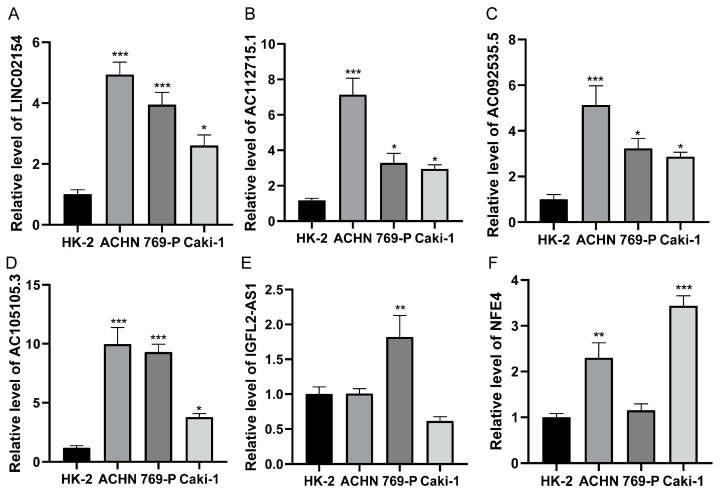
qRT-PCR for detection of lncRNA expression levels in a risk model in cancer cells (HK-2, ACHN, 769-P, and CAKI-1). (**A**) LINC02154; (**B**) AC112715.1; (**C**) AC092535.5; (**D**) AC105105.3; (**E**) IGFL2-AS1; (**F**) NFE4 in HK-2, ACHN, 769-P, and CAKI-1. * *p* < 0.05; ** *p* < 0.01; *** *p* < 0.001.

Compared with the normal renal cell line HK-2, LINC02154, AC112715.1, AC092535.5, and AC105105.3 were significantly upregulated in the renal cancer cell lines ACHN, 769-P, and CAKI-1 (Figure 12A–D). The expression of IGFL2-AS1 was only increased in the renal carcinoma cell line 769-P (Figure 12E), while the expression of NFE4 was increased in the renal carcinoma cell lines ACHN and CAKI-1 (Figure 12F).

### 3.10. Expression of Risk Model lncRNAs in Renal Cancer Tissue and Functional Verification

**Figure 13:**
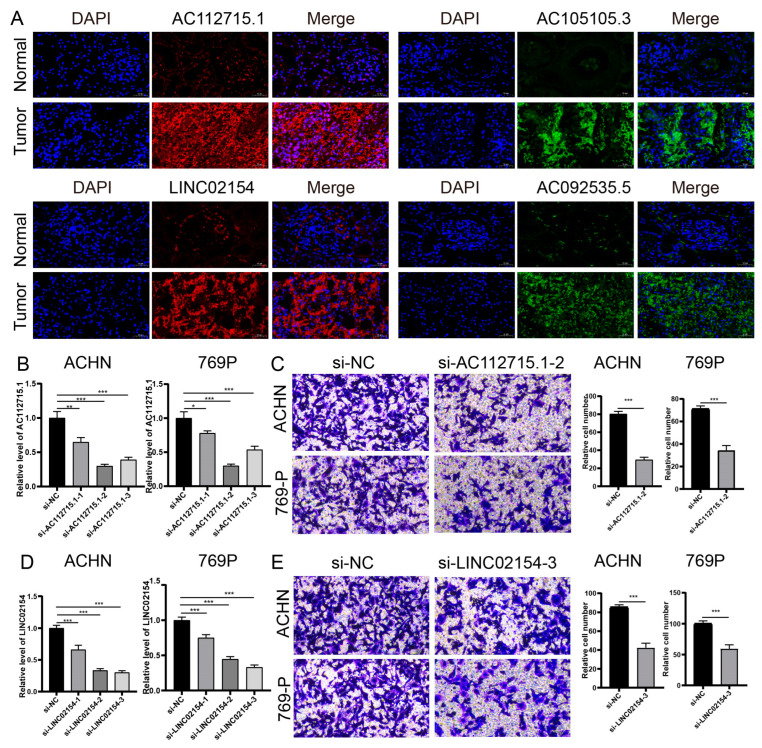
Verification of the expression of model lncRNA in renal cancer tissues and exploration of the impact on the invasion ability of renal cancer cell lines. (**A**) RNA FISH shows that AC112715.1, LINC02154, AC092535.5, and AC105105.3 are highly expressed in renal cancer tissues. (**B**) Effect of si-AC112715.1 on AC112715.1 expression in ACHN and 769P cells. (**C**) Effect of AC112715.1 knockdown on the invasion ability of ACHN and 769P cells. (**D**) Effect of si-LINC02154 on LINC021541 expression in ACHN and 769P cells. (**E**) Effect of LINC0215 knockdown on the invasion ability of ACHN and 769P cells. * *p* < 0.05; ** *p* < 0.01; *** *p* < 0.001.

We used RNA FISH technology to find out where LINC02154, AC112715.1, AC092535.5, and AC105105.3 were expressed and where they were located in kidney cancer tissue chips (Figure 13A). We found that these four lncRNAs are highly expressed in renal cancer tissues. AC112715.1 is mostly found in the nucleus, while INC02154, AC092535.5, and AC105105.3 are mostly found in the cytoplasm. We used si-LINC02154 and si-AC112715.1 to knock down the expression of LINC02154 and AC112715.1 in ACHN and 769P, respectively. Among them, si-AC112715.1-2 and si-LINC02154-3 had the best knockdown effects (Figure 13B,D). Through invasion experiments, we found that knocking down LINC02154 and AC112715.1 could inhibit the invasion ability of renal cancer cells (Figure 13C,E).

## 4. Discussion

During the development of kidney cancer, cancer cells usually penetrate the renal tubular epithelial cell layer and the corresponding basement membrane and enter the deep tissue of the kidney [21]. They may then begin to spread to surrounding tissue or spread further to other sites through the blood vessels or lymphatic system. The basement membrane typically deteriorates and ruptures as a result of renal cancer cell invasion [22]. This disruption helps cancer cells penetrate the basement membrane and enter deeper tissues [23]. Therefore, studying the integrity and recovery capabilities of the basement membrane is important for understanding the development and treatment of renal cancer.

Recent research indicates that lncRNAs are involved in various tumor genesis mechanisms, including carcinogenesis, proliferation, metastasis, migration, invasion, and angiogenesis [24]. Studies have shown that SNHG6 interacts with YBX1 to enhance the translation of HIF1, ultimately promoting ccRCC development and metastasis [25]. According to research, MRCCAT1 is an important lncRNA that promotes metastasis in ccRCC by suppressing NPR3 and promoting p38-MAPK signaling [26]. Song et al. reported that elevated expression of lncRNA ATB may promote renal cell cancer via binding to DNMT1, downregulating P53 and inhibiting the proliferation and migration of apoptotic cells [27]. However, basement membrane-associated lncRNAs in ccRCC have never been studied. Our team created a signature of the lncRNAs associated with basement membranes to predict the survival of ccRCC patients and validated the expression of risk model lncRNAs at the cellular level.

We discovered 784 basement membrane lncRNAs related to prognosis via differential and co-expression studies. According to univariate, LASSO, and multivariate Cox regression analyses, we found six basement membrane-associated lncRNAs (LINC02154, IGFL2-AS1, NFE4, AC112715.1, AC092535.5, and AC105105.3) that were substantially related to survival. Using the lncRNAs outlined before, we developed a risk model of basement membrane-associated lncRNAs to estimate the survival of ccRCC patients, and these lncRNAs are significantly expressed in kidney tissue cancer. LINC02154, AC112715.1, AC092535.5, and AC105105.3 were all significantly upregulated in three renal cancer cell lines, while the expression of IGFL2-AS1 and NFE4 was elevated in some renal cancer cell lines. We used RNA FISH to confirm that LINC02154, AC112715.1, AC092535.5, and AC105105.3 are highly expressed in renal cancer tissues. This is basically consistent with their expression levels in TCGA data.

LINC02154 increases liver cancer cell growth and spread by increasing SPC24 promoter activity and modulating the PI3K-AKT signaling pathway [28]; it may also be used to estimate the prognosis of laryngeal squamous cell carcinoma [29]. Studies have shown that renal cancer patients with high expression of LINC02154 have a poor prognosis, and knocking down LINC02154 can inhibit the invasion ability of cancer cells, which is consistent with our experimental results [30,31]. IGFL2-AS1 exerts a tumor-promoting effect in TSCC through the Wnt/β-catenin pathway and enhances the development of tongue squamous cell carcinoma [32]. KLF5’s proliferative and pro-survival effects are mediated by IGFL2-AS1 [33], and KLF5 promotes basal-like breast cancer by upregulating the expression of IGFL1 and cell growth and survival [34,35,36]. In renal cancer cells, IGFL2-AS1 contributes to the development of drug resistance in cancer cells [37,38]. According to ccRCC studies, patients who had a high level of lncRNA NEF4 expression had a shorter OS; this may be an independent prognostic factor for ccRCC [39]. Recently made available to the public are the last three lncRNAs (AC112715.1, AC092535.5, and AC105105.3). Specifically, these newly discovered basement membrane-associated lncRNAs could help us better comprehend ccRCC and explore innovative cancer therapy strategies.

Risk score prognosis accuracy was validated using the ROC and C-index. The risk score is an independent prognostic risk factor, as established via multivariate Cox analyses. The prognosis of patients with ccRCC was then predicted using a nomogram, and the calibration curve demonstrates that the actual measurements and projected values correspond well. Heatmaps of predictive and clinicopathological characteristics of basement membrane-associated lncRNAs demonstrated a correlation between risk scores and N stage, M stage, TMN stage, and grade. PCA study results show that risk model lncRNAs can better distinguish patients from two risk groups. The GO enrichment study indicates that binding activation of receptor, ligand, and cytokine signaling is tightly connected with basement membrane-associated lncRNAs. KEGG analysis revealed the cancer-related signaling pathways NF-kappa B pathway, TNF pathway, TGF-β pathway, and PI3K-Akt pathway and viral carcinogenesis were significantly active in basement membrane-associated lncRNAs. The TGF-β signaling pathway plays a crucial role in epithelial-mesenchymal transition (EMT) and cancer-associated fibroblast (CAF) production, and it has a significant influence on the progression of cancer [40]. In inflammatory-stimulated mammary epithelial cells, the long non-coding RNA NKILA may form a stable complex with NF-B/IκB and inhibit excessive activation of the NF-κB pathway [41]. Numerous studies have shown that the PI3K/Akt signaling pathway is inappropriately active in cancer and promotes tumor formation [42]. These findings indicate that basement membrane-associated lncRNAs play a crucial role in cancer formation.

Using multiple approaches to evaluate immune cell infiltration, we discovered substantial disparities between the two risk categories. Moreover, ssGSEA data demonstrated that the Type II IFN response is dormant in high-risk populations, whereas checkpoint and T cell co-inhibition is active. These findings indicate that the risk model lncRNAs may be active in the tumor immune microenvironment, working through immunological checkpoints, T cell co-inhibition, and Type II IFN response inactivation to suppress immune responses and promote ccRCC development [43].

Immune checkpoints comprise programmed death receptors and their corresponding ligands. T cells express programmed death receptors on their surfaces, while tumor cells express ligands [44]. The combination of the programmed death receptor and its ligand can exhaust T cells and render them incapable of killing tumor cells normally, allowing tumor cells to evade the host’s immune surveillance [43]. Therefore, the prognosis is worse for cancer patients with active immune checkpoints. In the high-risk group, the majority of immune checkpoints are active, the patients’ anti-tumor immunity is suppressed, and the patients have a dismal prognosis.

TIDE is used to evaluate the clinical response of ICI-treated patients. Higher TIDE scores represent a greater probability of immunological evasion, suggesting reduced responses and shorter survival in ICI-treated patients. High-risk individuals showed higher TIDE scores, indicating that they may respond less well to ICI therapy [45]. High-risk patients had higher mortality rates, and dead patients had a higher risk score. The high-risk category had greater rates of death, and those who passed away had higher risk scores. In addition, high-risk patients had shorter disease-free survival. We predict a number of drugs that may be therapeutically useful for kidney cancer and the susceptibility of individuals in two risk categories regarding these drugs. The prediction of patient sensitivity to drugs will help individualize chemotherapy drug selection for kidney cancer patients.

We created a basement membrane-associated lncRNA risk model in order to accurately forecast the prognosis of ccRCC patients using bioinformatics. Despite the fact that we have used a variety of techniques to improve our model, it still has a few flaws. The model is constructed based on the TCGA database. This model requires external datasets for validation and cannot avoid potential selection bias. Furthermore, to predict the prognostic value of BM-related lncRNAs, we utilized only data from public databases. We can only infer the effect of BM-related lncRNA on ccRCC based on limited clinical information, disregarding environmental and genetic variables. Lastly, we did not verify the biological functions of all risk model lncRNAs, and the mechanisms underlying their effects on ccRCC remain unknown. We will address these deficiencies in future research. We will refine and verify the role of basement membrane-associated lncRNAs in the future by collecting further clinical and experimental data.

We found that the BM-related risk model can well predict the prognosis of ccRCC patients and guide clinical treatment. The nomogram we constructed can predict the patient’s 1-, 3-, and 5-year survival rates using the patient’s risk score and clinical information. The BM-related risk model can divide ccRCC patients into high-risk and low-risk groups. People in the high-risk group had shorter survival times than those in the low-risk group. The risk model can be used to predict patient sensitivity to chemotherapy drugs. Based on the differences in sensitivity between high- and low-risk groups to different chemotherapy drugs, drugs can be selected according to patients’ sensitivity to guide their clinical treatment. Our results may provide fresh insight on patient outcome prediction and personalized therapy.

## 5. Conclusions

Our research reveals that the basement membrane-associated lncRNA risk model may accurately assess the prognosis of individuals with ccRCC. Moreover, this research may provide insight into the development of novel therapies for ccRCC.

## Data Availability

This research examines publicly accessible datasets. This information is available here: Data accessible via TCGA (https://tcga-data.nci.nih.gov/tcga/ (accessed on 1 December 2022)) and BM-BASE (https://bmbase.manchester.ac.uk (accessed on 3 December 2022)). Further details can be obtained from the corresponding author.

## References

[1] Vuong L., Kotecha R.R., Voss M.H., Hakimi A.A. (2019). Tumor Microenvironment Dynamics in Clear-Cell Renal Cell Carcinoma. Cancer Discov..

[2] Delman K.A. (2020). Introducing the “Virtual Tumor Board” series in CA: A Cancer Journal for Clinicians. CA Cancer J. Clin..

[3] Choueiri T.K., Motzer R.J. (2017). Systemic Therapy for Metastatic Renal-Cell Carcinoma. N. Engl. J. Med..

[4] Lalani A.A., McGregor B.A., Albiges L., Choueiri T.K., Motzer R., Powles T., Wood C., Bex A. (2019). Systemic Treatment of Metastatic Clear Cell Renal Cell Carcinoma in 2018: Current Paradigms, Use of Immunotherapy, and Future Directions. Eur. Urol..

[5] Jonasch E., Walker C.L., Rathmell W.K. (2021). Clear cell renal cell carcinoma ontogeny and mechanisms of lethality. Nat. Rev. Nephrol..

[6] Napolitano L., Manfredi C., Cirillo L., Fusco G.M., Passaro F., Abate M., La Rocca R., Mastrangelo F., Spirito L., Pandolfo S.D. (2023). Cytoreductive Nephrectomy and Metastatic Renal Cell Carcinoma: State of the Art and Future Perspectives. Medicina.

[7] Boissier R., Hevia V., Bruins H.M., Budde K., Figueiredo A., Lledo-Garcia E., Olsburgh J., Regele H., Taylor C.F., Zakri R.H. (2018). The Risk of Tumour Recurrence in Patients Undergoing Renal Transplantation for End-stage Renal Disease after Previous Treatment for a Urological Cancer: A Systematic Review. Eur. Urol..

[8] Yurchenco P.D. (2011). Basement membranes: Cell scaffoldings and signaling platforms. Cold Spring Harb. Perspect. Biol..

[9] Chang J., Chaudhuri O. (2019). Beyond proteases: Basement membrane mechanics and cancer invasion. J. Cell Biol..

[10] Fidler A.L., Darris C.E., Chetyrkin S.V., Pedchenko V.K., Boudko S.P., Brown K.L., Gray Jerome W., Hudson J.K., Rokas A., Hudson B.G. (2017). Collagen IV and basement membrane at the evolutionary dawn of metazoan tissues. eLife.

[11] Siegel R.L., Miller K.D., Fuchs H.E., Jemal A. (2022). Cancer statistics, 2022. CA Cancer J. Clin..

[12] Quinn J.J., Chang H.Y. (2016). Unique features of long non-coding RNA biogenesis and function. Nat. Rev. Genet..

[13] Liu S.J., Dang H.X., Lim D.A., Feng F.Y., Maher C.A. (2021). Long noncoding RNAs in cancer metastasis. Nat. Rev. Cancer.

[14] Pandey G.K., Kanduri C. (2022). Long Non-Coding RNAs: Tools for Understanding and Targeting Cancer Pathways. Cancers.

[15] Liu P.F., Farooqi A.A., Peng S.Y., Yu T.J., Dahms H.U., Lee C.H., Tang J.Y., Wang S.C., Shu C.W., Chang H.W. (2022). Regulatory effects of noncoding RNAs on the interplay of oxidative stress and autophagy in cancer malignancy and therapy. Semin. Cancer Biol..

[16] Shan G., Huang T., Tang T. (2022). Long non-coding RNA MEG8 induced by PLAG1 promotes clear cell renal cell carcinoma through the miR-495-3p/G3BP1 axis. Pathol. Res. Pract..

[17] Zhang Z., Fu X., Gao Y., Nie Z. (2022). LINC01535 Attenuates ccRCC Progression through Regulation of the miR-146b-5p/TRIM2 Axis and Inactivation of the PI3K/Akt Pathway. J. Oncol..

[18] Barik G.K., Sahay O., Behera A., Naik D., Kalita B. (2021). Keep your eyes peeled for long noncoding RNAs: Explaining their boundless role in cancer metastasis, drug resistance, and clinical application. Biochim. Biophys Acta Rev. Cancer.

[19] Jayadev R., Morais M., Ellingford J.M., Srinivasan S., Naylor R.W., Lawless C., Li A.S., Ingham J.F., Hastie E., Chi Q. (2022). A basement membrane discovery pipeline uncovers network complexity, regulators, and human disease associations. Sci. Adv..

[20] Fang C., Liu S., Feng K., Huang C., Zhang Y., Wang J., Lin H., Wang J., Zhong C. (2022). Ferroptosis-related lncRNA signature predicts the prognosis and immune microenvironment of hepatocellular carcinoma. Sci. Rep..

[21] Morell-Quadreny L., Rubio J., Lopez-Guerrero J.A., Casanova J., Ramos D., Iborra I., Solsona E., Llombart-Bosch A. (2003). Disruption of basement membrane, extracellular matrix metalloproteinases and E-cadherin in renal-cell carcinoma. Anticancer Res..

[22] Majo S., Courtois S., Souleyreau W., Bikfalvi A., Auguste P. (2020). Impact of Extracellular Matrix Components to Renal Cell Carcinoma Behavior. Front. Oncol..

[23] Chen Y., Lu H., Tao D., Fan M., Zhuang Q., Xing Z., Chen Z., He X. (2017). Membrane type-2 matrix metalloproteinases improve the progression of renal cell cancer. Int. J. Clin. Exp. Pathol..

[24] Zhou J., Zhang Y., Li S., Zhou Q., Lu Y., Shi J., Liu J., Wu Q., Zhou S. (2020). Dendrobium nobile Lindl. alkaloids-mediated protection against CCl(4-)induced liver mitochondrial oxidative damage is dependent on the activation of Nrf2 signaling pathway. BioMedicine.

[25] Zhao P., Deng Y., Wu Y., Guo Q., Zhou L., Yang X., Wang C. (2021). Long noncoding RNA SNHG6 promotes carcinogenesis by enhancing YBX1-mediated translation of HIF1alpha in clear cell renal cell carcinoma. FASEB J..

[26] Li J.K., Chen C., Liu J.Y., Shi J.Z., Liu S.P., Liu B., Wu D.S., Fang Z.Y., Bao Y., Jiang M.M. (2017). Long noncoding RNA MRCCAT1 promotes metastasis of clear cell renal cell carcinoma via inhibiting NPR3 and activating p38-MAPK signaling. Mol. Cancer.

[27] Song C., Xiong Y., Liao W., Meng L., Yang S. (2019). Long noncoding RNA ATB participates in the development of renal cell carcinoma by downregulating p53 via binding to DNMT1. J. Cell. Physiol..

[28] Yue H., Wu K., Liu K., Gou L., Huang A., Tang H. (2022). LINC02154 promotes the proliferation and metastasis of hepatocellular carcinoma by enhancing SPC24 promoter activity and activating the PI3K-AKT signaling pathway. Cell. Oncol..

[29] Zhang G., Fan E., Zhong Q., Feng G., Shuai Y., Wu M., Chen Q., Gou X. (2019). Identification and potential mechanisms of a 4-lncRNA signature that predicts prognosis in patients with laryngeal cancer. Hum. Genom..

[30] Liu L., Zhuang M., Tu X.H., Li C.C., Liu H.H., Wang J. (2023). Bioinformatics analysis of markers based on m(6) A related to prognosis combined with immune invasion of renal clear cell carcinoma. Cell Biol. Int..

[31] Shen J., Wang L., Bi J. (2023). Bioinformatics analysis and experimental validation of cuproptosis-related lncRNA LINC02154 in clear cell renal cell carcinoma. BMC Cancer.

[32] Zhao R., Wang S., Tan L., Li H., Liu J., Zhang S. (2023). IGFL2-AS1 facilitates tongue squamous cell carcinoma progression via Wnt/beta-catenin signaling pathway. Oral Dis..

[33] Wang H., Shi Y., Chen C.H., Wen Y., Zhou Z., Yang C., Sun J., Du G., Wu J., Mao X. (2021). KLF5-induced lncRNA IGFL2-AS1 promotes basal-like breast cancer cell growth and survival by upregulating the expression of IGFL1. Cancer Lett..

[34] Tracy K.M., Tye C.E., Page N.A., Fritz A.J., Stein J.L., Lian J.B., Stein G.S. (2018). Selective expression of long non-coding RNAs in a breast cancer cell progression model. J. Cell. Physiol..

[35] Ma Y., Liu Y., Pu Y.S., Cui M.L., Mao Z.J., Li Z.Z., He L., Wu M., Wang J.H. (2020). LncRNA IGFL2-AS1 functions as a ceRNA in regulating ARPP19 through competitive binding to miR-802 in gastric cancer. Mol. Carcinog..

[36] Cen X., Huang Y., Lu Z., Shao W., Zhuo C., Bao C., Feng S., Wei C., Tang X., Cen L. (2021). LncRNA IGFL2-AS1 Promotes the Proliferation, Migration, and Invasion of Colon Cancer Cells and is Associated with Patient Prognosis. Cancer Manag. Res..

[37] Cheng B., Xie M., Zhou Y., Li T., Liu W., Yu W., Jia M., Yu S., Chen L., Dai R. (2023). Vascular mimicry induced by m(6)A mediated IGFL2-AS1/AR axis contributes to pazopanib resistance in clear cell renal cell carcinoma. Cell Death Discov..

[38] Pan Y., Lu X., Shu G., Cen J., Lu J., Zhou M., Huang K., Dong J., Li J., Lin H. (2023). Extracellular Vesicle-Mediated Transfer of LncRNA IGFL2-AS1 Confers Sunitinib Resistance in Renal Cell Carcinoma. Cancer Res..

[39] Pan Q., Wang L., Zhang H., Liang C., Li B. (2019). Identification of a 5-Gene Signature Predicting Progression and Prognosis of Clear Cell Renal Cell Carcinoma. Med. Sci. Monit..

[40] Peng D., Fu M., Wang M., Wei Y., Wei X. (2022). Targeting TGF-beta signal transduction for fibrosis and cancer therapy. Mol. Cancer.

[41] Liu B., Sun L., Liu Q., Gong C., Yao Y., Lv X., Lin L., Yao H., Su F., Li D. (2015). A cytoplasmic NF-kappaB interacting long noncoding RNA blocks IkappaB phosphorylation and suppresses breast cancer metastasis. Cancer Cell.

[42] He Y., Sun M.M., Zhang G.G., Yang J., Chen K.S., Xu W.W., Li B. (2021). Targeting PI3K/Akt signal transduction for cancer therapy. Signal Transduct. Target. Ther..

[43] Kalbasi A., Ribas A. (2020). Tumour-intrinsic resistance to immune checkpoint blockade. Nat. Rev. Immunol..

[44] Morad G., Helmink B.A., Sharma P., Wargo J.A. (2021). Hallmarks of response, resistance, and toxicity to immune checkpoint blockade. Cell.

[45] Jiang P., Gu S., Pan D., Fu J., Sahu A., Hu X., Li Z., Traugh N., Bu X., Li B. (2018). Signatures of T cell dysfunction and exclusion predict cancer immunotherapy response. Nat. Med..

